# Relationship between screen time and nutrient intake in Japanese children and adolescents: a cross-sectional observational study

**DOI:** 10.1186/s12199-018-0725-0

**Published:** 2018-08-07

**Authors:** Hiromasa Tsujiguchi, Daisuke Hori, Yasuhiro Kambayashi, Toshio Hamagishi, Hiroki Asakura, Junko Mitoma, Masami Kitaoka, Enoch Olando Anyenda, Thao Thi Thu Nguyen, Yohei Yamada, Koichiro Hayashi, Tadashi Konoshita, Takiko Sagara, Aki Shibata, Satoshi Sasaki, Hiroyuki Nakamura

**Affiliations:** 1 Department of Environmental and Preventive Medicine, Graduate School of Medical Sciences, Kanazawa University, 13-1 Takaramachi, Kanazawa, Ishikawa Japan; 2 Third Department of Internal Medicine, University of Fukui Faculty of Medical Sciences, 23-3 Matsuokashimoaiduki, Eiheiji, Fukui, Japan; 3 Department of Social and Preventive Epidemiology, School of Public Health, The University of Tokyo, 7-3-1 Hongo, Bunkyoku, Tokyo, Japan

**Keywords:** Screen, Television, Nutrients, Children, Adolescents

## Abstract

**Background:**

Sedentary behaviors have recently become an important public health issue. We aimed to investigate the relationship between screen time and nutrient intake in children and adolescents.

**Methods:**

The present study was conducted in 2013. Data were collected from children and adolescents aged between 6 and 15 years old in Shika town. Questionnaires were distributed to 1459 subjects, 1414 of whom participated in the study (96.9%). Sedentary behaviors were assessed based on participants’ screen behaviors (television (TV) viewing, personal computer (PC) use, and mobile phone (MP) use). The main outcomes were the intake of nutrients from a validated food frequency questionnaire. Analysis of covariance (ANCOVA) was used to examine the significance of differences in nutrient intake estimates. Multivariate linear regression analyses, adjusting for age, BMI, and physical activity, were used to provide parameter estimates (*β*) and 95% CI for the relationship between screen time and nutrient intake.

**Results:**

In boys, longer TV viewing times correlated or tended to correlate with a lower intake of protein, potassium, calcium, iron, vitamin K, vitamin B-2, and total dietary fiber. In girls, longer TV viewing times correlated with a lower intake of protein, sodium, calcium, vitamin D, and vitamin B-2. Longer TV viewing times correlated with a higher intake of n-6 fatty acids in girls. PC use was related or tended to be related to a lower intake of potassium, iron, vitamin K, and folic acid in boys, but not in girls. A relationship was observed between MP use and a lower intake of vitamin K in boys, and MP use and a higher intake of vitamin D in girls.

**Conclusions:**

The present results revealed that longer TV viewing times are associated with less protein, minerals, vitamins, and total dietary fiber intake in children and adolescents. It was also revealed that boys with PC use have less minerals and vitamins. These results support the need to design intervention programs that focus on decreasing TV viewing time in both sexes and PC use in boys while encouraging adherence to dietary guidelines among children and adolescents.

## Background

Sedentary behaviors have recently become an important public health issue. Sedentary behaviors are “sitting or lying tasks performed in the waking hours with low levels of energy expenditure” [1]. There is evidence to suggest that sedentary behaviors are linked to adverse health outcomes. Observational studies reported that a prolonged sedentary time, in particular time spent watching television (TV), was linked to an increased risk of obesity, type 2 diabetes mellitus, cardiovascular disease, and premature death [2, 3]. However, these relationships are not always consistent across age, gender, or health outcomes.

Although sedentary behaviors have been suggested to contribute to young individuals being overweight and obese, the evidence obtained to support this remains controversial. This may be due to the influence of a third variable or co-existing health behaviors. One variable that may co-vary with sedentary behaviors is diet [4, 5]. The consumption of a healthier diet reduces the risk of obesity and numerous chronic diseases [6].

Previous studies demonstrated that TV viewing was linked to a less healthy diet such as a lower intake of fruit and vegetables; the higher consumption of energy-dense snacks, drinks, and fast food; and a higher total energy intake in young individuals [4, 5]. These findings suggest that dietary intake plays a role in the relationship between TV viewing and health [7]. However, the relationships among sedentary behaviors and nutrient intake have not been examined in sufficient detail in children and adolescents.

Most of the published research on sedentary and dietary behaviors has focused on TV viewing times [4, 5]. Due to the recent increase in non-TV screen times, more information is needed on the relationship between personal computer (PC) and mobile phone (MP) use and nutrient intake in children and adolescents.

Therefore, we herein aimed to investigate the relationship between screen time, including PC and MP use, and nutrient intake among children and adolescents. To the best of our knowledge, few studies have examined this relationship.

## Methods

### Data and participants

We utilized cross-sectional data from the Shika study in Shika town. Shika town is located in a rural area of Ishikawa prefecture, Japan, with a population of 21,061 individuals. Major industries include electronic component manufacturing, farming, construction, and tourism. The climate is humid subtropical. The Shika study is an ongoing population-based survey that aims to investigate the lifestyle and health status of the Japanese population. This cross-sectional study was primarily designed to assess nutrient intake and associated behaviors in a sample of Japanese children and adolescents. The present study was performed between October and December, 2013. Data were collected from the guardians of children aged between 6 and 15 years old in Shika town. Our laboratory signed a research agreement with Shika town for health promotion. All the students of compulsory school age who went to Elementary School or Junior High School in Shika town in 2013 were eligible to participate in the study. We distributed questionnaires in collaboration with these Schools. We visited each school and handed questionnaires to the school principal or the school nurse. The school principal or the school nurse handed them to the homeroom teacher. The homeroom teacher explained about questionnaires and handed them to students. Questionnaires were distributed to 1459 guardians through students, 1414 of whom participated in this study (96.9%). We asked the guardians to fill in the questionnaire with their children, as much as they could, to reflect their children’s usual behavior accurately, to the greatest extent possible.

### Primary measures

#### Screen time (independent variable)

Sedentary behaviors were assessed based on the screen behavior of participants during the week. Screen behavior was assessed by asking about the time spent on each screen on an average school day. We categorized TV viewing times as moderate (< 2 h/day) and higher (≧ 2 h/day). This is consistent with the definitions of high and low TV viewing in the literature [8]. PC and MP use were categorized as non-users and users. When the participants viewed DVDs on PC or channel on YouTube, they counted such viewing times as PC using times, not as TV viewing times.

#### Dietary variables (dependent variables)

The main outcome was the intake of nutrients from the validated food frequency questionnaire. We utilized the brief self-administered dietary history questionnaire (BDHQ). BDHQ asks about dietary history during the preceding 1 month. BDHQ is based on a comprehensive version of a validated self-administered questionnaire (i.e., DHQ) [9]. BDHQ 3y, 10y, and 15y (for Japanese preschool children aged 3–6 years, elementary school children aged 7–12 years, and junior high school children aged 13–15 years, respectively) were developed by modifying the adult version. We used BDHQ 10y for the participants of elementary school and 15y for the participants of junior high school). Each BDHQ listed 54 to 67 food items, respectively, in view of the legibility and knowledge of food. The intake of energy and 99 nutrients was estimated using a computer algorithm for BDHQ. Nutrient intake was reported in terms of energy density (% energy or per 1000 kcal). Its validity is currently moderate to low [10, 11]. Therefore, we selected nutrients that were shown to have moderate validity in the study. Details on BDHQ are described elsewhere [9, 12–14]. Participants who reported less than 600 kcal or more than 4000 kcal energy intake per day were excluded from the analyses.

#### Covariates (demographics, body weight status, and physical activity)

Covariates included in the multivariable analysis consisted of age, body weight status, and physical activity (PA). Obese children are known to be more strongly affected by TV, with respect to their food intake, than children who are a normal weight or overweight [15]. Body weight status was based on body mass index (BMI) calculated from self-reported heights and weights. BMI was computed based on the standard formula (kg/m^2^) [6]. BMI was used to classify children as underweight, normal weight, and overweight (including obese) based on the International Obesity Task Force reference [16]. Although participation in sedentary behaviors and PA occur independently [17], research suggests that both types of PA-related behaviors may be associated with dietary behavior [2, 4]. The questionnaire was used to assess the level of PA, which was evaluated by asking the time spent doing PA on an average school day.

### Statistical analysis

All analyses were stratified by sex. Descriptive statistics were used to describe participants’ demographics, health behavior, and nutrient intake. Categorical variables were presented as percentages (%) and frequencies (*N*). Continuous variables were summarized as the mean and standard deviation (SD). Analysis of covariance (ANCOVA), adjusting for age, body weight status, and PA, on screen time was used to examine significant differences in nutrient intake estimates. Multivariate linear regression analyses, adjusting for age, BMI, and PA, were used to provide parameter estimates (β) and 95% confidence interval (CI) for the relationship between screen time and nutrient intake. Statistical Package for Social Science (SPSS) version 19 was used for these analyses. Statistical tests were two-sided at a significance level of 5%.

## Results

### Gender differences in the prevalence of screen time, body weight status, PA, and nutrient intake

The characteristics of the studied population are shown in Table 1. Thirty-one boys and nine girls were excluded from the analyses. A total of 1374 participants provided data for the variables of interest and were included in the analyses. Boys accounted for 49.4% (*N* = 679) of samples, while 50.6% (*N* = 695) were girls. The average ages of the participants were 10.72 (SD = 2.61) years for boys and 10.69 (SD = 2.65) years for girls. The rate of participants who were overweight was significantly higher in boys (*p* < 0.001). The rates of participants using MP (*p* < 0.001) and being physically inactive (*p* < 0.001) were significantly higher in girls. Boys were more likely to have a total energy intake (*p* < 0.001) and consume carbohydrates (*p* < 0.001). Girls were more likely to consume protein (*p* = 0.027), fat (*p* < 0.001), fatty acids (saturated fatty acids, *p* = 0.002; n-3 fatty acids, *p* = 0.010; n-6 fatty acids, *p* < 0.001), minerals (excluding calcium; sodium, *p* < 0.001; potassium, *p* < 0.001; iron, *p* < 0.001), vitamins (excluding vitamin D; vitamin K, *p* < 0.001; vitamin B-1, *p* = 0.016; vitamin B-2, *p* = 0.003; folic acid, *p* < 0.001; vitamin C, *p* < 0.001), cholesterol (*p* < 0.001), and total dietary fiber (*p* < 0.001).

**Table 1:** The characteristics of the studied population

	Boys	Girls	*p* ^†^
Number (*N* (%))	679 (49.4%)	695 (50.6%)	
Age (average (SD))	10.72 (2.61)	10.69 (2.65)	0.843
Body mass index (BMI) categories (*N* (%))			
Underweight	39 (5.7%)	55 (7.9%)	< 0.001
Normal weight	484 (71.3%)	545 (78.4%)	
Overweight	156 (23.0%)	95 (13.7%)	
Physical activity (*N* (%))			
< 1 h	324 (48.5%)	424 (62.2%)	< 0.001
1–3 h	284 (42.5%)	221 (32.4%)	
> 3 h	60 (9.0%)	37 (5.4%)	
Television (TV) viewing time (*N* (%))			
< 2 h	390 (58.6%)	388 (56.6%)	0.456
> 2 h	275 (41.4%)	297 (43.4%)	
Personal computer (PC) using (*N* (%))			
Non-user	441 (66.3%)	454 (66.4%)	0.982
User	224 (33.7%)	230 (33.6%)	
Mobile phone (MP) using (*N* (%))			
Non-user	509 (76.7%)	465 (68.1%)	< 0.001
User	155 (23.3%)	218 (31.9%)	
Nutrients intake (average (SD))			
Energy (kcal)	1953.04 (669.45)	1676.75 (568.94)	< 0.001
Protein (%E)	13.95 (2.31)	14.22 (2.28)	0.027
Fat (%E)	27.82 (5.30)	29.10 (5.13)	< 0.001
Carbohydrate (%E)	56.61 (6.57)	55.30 (6.32)	< 0.001
Saturated fatty acids (%E)	9.17 (2.20)	9.55 (2.22)	0.002
n-3 fatty acids (%E)	1.03 (0.29)	1.07 (0.29)	0.010
n-6 fatty acids (%E)	4.88 (1.15)	5.14 (1.08)	< 0.001
Sodium (mg/1000 kcal)	2279.26 (537.76)	2418.16 (541.57)	< 0.001
Potassium (mg/1000 kcal)	1171.14 (276.87)	1237.82 (267.08)	< 0.001
Calcium (mg/1000 kcal)	353.40 (113.29)	363.69 (97.47)	0.071
Iron (mg/1000 kcal)	3.54 (0.73)	3.81 (0.73)	< 0.001
Vitamin D (μg/1000 kcal)	5.01 (2.90)	5.08 (2.87)	0.674
Vitamin K (μg/1000 kcal)	106.18 (52.75)	115.91 (55.07)	0.001
Vitamin B-1 (mg/1000 kcal)	0.40 (0.07)	0.41 (0.08)	0.016
Vitamin B-2 (mg/1000 kcal)	0.70 (0.19)	0.73 (0.16)	0.003
Folic acid (μg/1000 kcal)	141.11 (44.77)	154.88 (44.64)	< 0.001
Vitamin C (mg/1000 kcal)	49.85 (22.18)	55.24 (22.78)	< 0.001
Cholesterol (mg/1000 kcal)	165.89 (54.26)	181.55 (61.06)	< 0.001
Total dietary fiber (g/1000 kcal)	5.33 (1.34)	5.77 (1.32)	< 0.001

^†^*t* tests for continuous variables and *χ*^2^ tests for categorical variables

### Nutrients intake according to screen time

There was a significant main effect of screen time on the consumption of nutrients. The following screen time correlated or tended to correlate with nutrient intake in ANCOVA (Tables 2, 3, and 4). The intake of protein (*p* = 0.005), minerals (excluding sodium; potassium, *p* = 0.064; calcium, *p* = 0.005; iron, *p* = 0.034), vitamin K (*p* = 0.018), vitamin B-2 (*p* = 0.050), and total dietary fiber (*p*2 = 0.082) was lower in boys with longer TV viewing times. The intake of protein (*p*2 = 0.027), sodium (*p* = 0.071), calcium (*p* < 0.001), vitamin D (*p* = 0.079), and vitamin B-2 (*p* = 0.032) was lower in girls with longer TV viewings. The intake of n-6 fatty acids was higher (*p* = 0.028) in girls with longer TV viewings.

**Table 2:** The nutrients intake according to television (TV) viewing time

	Boys			Girls		
	< 2 h (*N* = 390)	> 2 h (*N* = 274)	*p* ^†^	< 2 h (*N* = 387)	> 2 h (*N* = 295)	*p* ^†^
Age (average (CI))	10.64 (10.37–10.90)	10.86 (5.90–15.82)	< 0.001	10.42 (10.13–10.71)	11.06 (10.80–11.31)	< 0.001
BMI categories (*N* (%))						
Underweight	27 (6.9%)	11 (4.0%)	0.008	33 (8.5%)	22 (7.4%)	0.113
Normal weight	289 (74.1%)	186 (67.6%)		311 (80.2%)	225 (75.8%)	
Overweight	74 (19.0%)	77 (28.4%)		43 (11.3%)	48 (16.8%)	
Physical activity (*N* (%))						
< 1 h	190 (48.7%)	131 (47.8%)	0.085	256 (66.1%)	168 (56.9%)	0.049
1–3 h	158 (40.5%)	126 (46.0%)		112 (28.9%)	109 (36.9%)	
> 3 h	42 (10.8%)	17 (6.2%)		19 (4.9%)	18 (6.1%)	
Nutrition (average (CI)^††^)						
Protein (%E)	14.20 (13.86–14.55)	13.69 (13.30–14.09)	0.005	14.20 (13.82–14.58)	13.81 (13.43–14.20)	0.027
Fat (%E)	27.71 (26.91–28.52)	27.46 (26.55–28.38)	0.550	28.70 (27.83–29.56)	29.07 (28.18–29.95)	0.358
Carbohydrate (%E)	56.51 (55.50–57.51)	57.27 (56.13–58.41)	0.146	55.67 (54.60–56.73)	55.75 (54.66–56.84)	0.868
Saturated fatty acids (%E)	9.06 (8.73–9.40)	9.05 (8.67–9.43)	0.940	9.48 (9.11–9.85)	9.38 (9.00–9.76)	0.564
n-3 fatty acids (%E)	1.04 (1.00–1.09)	1.01 (0.96–1.06)	0.206	1.08 (1.03–1.13)	1.10 (1.05–1.15)	0.328
n-6 fatty acids (%E)	4.89 (4.72–5.05)	4.79 (4.61–4.98)	0.265	5.05 (4.87–5.23)	5.23 (5.05–5.42)	0.028
Sodium (mg/1000 kcal)	2249.64 (2174.33–2324.95)	2199.62 (2114.20–2285.04)	0.203	2379.62 (2294.17–2465.08)	2307.88 (2220.69–2395.07)	0.071
Potassium (mg/1000 kcal)	1218.28 (1176.04–1260.53)	1177.53 (1129.61–1225.44)	0.064	1238.57 (1194.36–1282.78)	1208.09 (1162.99–1253.20)	0.137
Calcium (mg/1000 kcal)	363.39 (346.14–380.65)	338.28 (318.71–357.85)	0.005	375.46 (359.21–391.72)	341.20 (324.61–357.78)	< 0.001
Iron (mg/1000 kcal)	3.65 (3.54–3.76)	3.53 (3.41–3.66)	0.034	3.79 (3.66–3.91)	3.73 (3.60–3.86)	0.307
Vitamin D (μg/1000 kcal)	5.18 (4.74–5.61)	4.94 (4.44–5.43)	0.286	5.41 (4.94–5.89)	5.03 (4.54–5.51)	0.079
Vitamin K (μg/1000 kcal)	118.66 (110.67–126.64)	108.78 (99.72–117.83)	0.018	117.10 (107.79–126.41)	114.01 (104.51–123.50)	0.473
Vitamin B-1 (mg/1000 kcal)	0.41 (0.40–0.42)	0.40 (0.39–0.41)	0.123	0.41 (0.39–0.42)	0.40 (0.39–0.41)	0.285
Vitamin B-2 (mg/1000 kcal)	0.73 (0.70–0.75)	0.70 (0.67–0.73)	0.050	0.74 (0.71–0.76)	0.71 (0.68–0.74)	0.032
Folic acid (μg/1000 kcal)	149.39 (142.58–156.20)	143.56 (135.83–151.28)	0.101	153.11 (145.69–160.52)	152.00 (144.44–159.57)	0.749
Vitamin C (mg/1000 kcal)	52.52 (49.12–55.92)	51.90 (48.04–55.76)	0.725	54.68 (50.95–58.42)	56.32 (52.50–60.13)	0.346
Cholesterol (mg/1000 kcal)	168.10 (159.83–176.37)	161.09 (151.71–170.47)	0.104	175.68 (165.70–185.65)	177.43 (167.25–187.61)	0.705
Total dietary fiber (g/1000 kcal)	5.64 (5.44–5.84)	5.46 (5.24–5.69)	0.082	5.72 (5.50–5.95)	5.65 (5.43–5.88)	0.499

^†^*χ*^2^ tests for categorical variables and analysis of covariances for continuous variables, adjusting for age, BMI, and physical activity

^††^Adjusted mean and standard errors calculated by ANCOVA

**Table 3:** The nutrients intake according to personal computer (PC) using

	Boys			Girls		
	Non-user (*N* = 440)	User (*N* = 224)	*p* ^†^	Non-user (*N* = 452)	User (*N* = 228)	*p* ^†^
Age (average (CI))	10.26 (10.02–10.51)	11.67 (11.35–11.99)	< 0.001	10.05 (9.80–10.29)	11.97 (11.69–12.25)	< 0.001
BMI categories (*N* (%))						
Underweight	27 (6.1%)	11 (4.9%)	0.004	35 (7.7%)	19 (8.3%)	0.024
Normal weight	329 (74.6%)	144 (64.3%)		345 (76.0%)	190 (83.0%)	
Overweight	84 (19.3%)	69 (30.8%)		72 (16.3%)	19 (8.7%)	
Physical activity (*N* (%))						
< 1 h	228 (51.8%)	93 (41.5%)	0.039	310 (68.6%)	114 (50.0%)	< 0.001
1–3 h	177 (40.2%)	107 (47.8%)		122 (27.0%)	97 (42.5%)	
> 3 h	35 (8.0%)	24 (10.7%)		20 (4.4%)	17 (7.5%)	
Nutrition (average (CI)^††^)						
Protein (%E)	14.09 (13.74–14.45)	13.84 (13.44–14.25)	0.206	14.05 (13.68–14.42)	13.97 (13.55–14.40)	0.702
Fat (%E)	27.67 (26.86–28.49)	27.50 (26.57–28.42)	0.696	28.75 (27.91–29.59)	29.14 (28.18–30.11)	0.376
Carbohydrate (%E)	56.66 (55.64–57.68)	57.07 (55.92–58.23)	0.461	55.81 (54.77–56.85)	55.47 (54.28–56.66)	0.537
Saturated fatty acids (%E)	9.07 (8.72–9.41)	9.07 (8.68–9.46)	0.997	9.36 (9.00–9.72)	9.60 (9.19–10.02)	0.205
n-3 fatty acids (%E)	1.04 (0.99–1.09)	1.01 (0.96–1.06)	0.189	1.09 (1.04–1.13)	1.10 (1.05–1.16)	0.514
n-6 fatty acids (%E)	4.89 (4.72–5.05)	4.77 (4.59–4.96)	0.212	5.15 (4.97–5.32)	5.11 (4.91–5.31)	0.691
Sodium (mg/1000 kcal)	2249.19 (2172.49–2325.89)	2195.30 (2108.20–2282.40)	0.203	2350.55 (2267.11–2433.99)	2348.09 (2252.22–2443.95)	0.956
Potassium (mg/1000 kcal)	1218.94 (1175.90–1261.98)	1173.58 (1124.70–1222.46)	0.056	1229.86 (1186.70–1273.02)	1214.39 (1164.80–1263.98)	0.501
Calcium (mg/1000 kcal)	357.02 (339.36–374.68)	348.61 (328.56–368.66)	0.388	359.23 (343.16–375.30)	360.91 (342.44–379.37)	0.845
Iron (mg/1000 kcal)	3.65 (3.53–3.76)	3.53 (3.40–3.66)	0.060	3.77 (3.65–3.89)	3.74 (3.60–3.88)	0.658
Vitamin D (μg/1000 kcal)	5.02 (4.58–5.46)	5.19 (4.69–5.69)	0.479	5.15 (4.69–5.61)	5.43 (4.90–5.96)	0.253
Vitamin K (μg/1000 kcal)	118.36 (110.22–126.49)	108.52 (99.29–117.76)	0.029	115.79 (106.72–124.86)	114.55 (104.12–124.97)	0.797
Vitamin B-1 (mg/1000 kcal)	0.41 (0.40–0.42)	0.40 (0.39–0.42)	0.355	0.41 (0.39–0.42)	0.40 (0.38–0.41)	0.197
Vitamin B-2 (mg/1000 kcal)	0.72 (0.69–0.75)	0.70 (0.67–0.74)	0.284	0.72 (0.70–0.75)	0.72 (0.69–0.75)	0.873
Folic acid (μg/1000 kcal)	149.92 (142.99–156.85)	142.32 (134.46–150.19)	0.047	153.37 (146.15–160.60)	150.46 (142.16–158.76)	0.449
Vitamin C (mg/1000 kcal)	53.08 (49.63–56.53)	50.85 (46.93–54.77)	0.241	55.74 (52.09–59.38)	54.61 (50.42–58.80)	0.561
Cholesterol (mg/1000 kcal)	164.83 (156.41–173.24)	165.98 (156.42–175.54)	0.804	175.17 (165.44–184.89)	179.43 (168.26–190.60)	0.410
Total dietary fiber (g/1000 kcal)	5.62 (5.42–5.82)	5.47 (5.24–5.70)	0.181	5.69 (5.48–5.91)	5.67 (5.42–5.92)	0.855

^†^*χ*^2^ tests for categorical variables and analysis of covariances for continuous variables, adjusting for age, BMI, and physical activity

^††^Adjusted mean and standard errors calculated by ANCOVA

**Table 4:** The nutrients intake according to mobile phone (MP) using

	Boys			Girls		
	Non-user (*N* = 508)	User (*N* = 155)	*p* ^†^	Non-user (*N* = 462)	User (*N* = 217)	*p* ^†^
Age (average (CI))	10.33 (10.12–10.55)	12.08 (11.69–12.47)	< 0.001	9.99 (9.76–10.23)	12.21 (11.92–12.49)	< 0.001
BMI categories (*N* (%))						
Underweight	28 (5.5%)	10 (6.5%)	0.901	38 (8.2%)	16 (7.3%)	0.211
Normal weight	363 (71.3%)	110 (71.0%)		356 (76.6%)	178 (82.1%)	
Overweight	117 (23.2%)	35 (22.6%)		68 (15.3%)	23 (10.6%)	
Physical activity (*N* (%))						
< 1 h	263 (51.8%)	60 (38.7%)	0.017	320 (69.3%)	103 (47.5%)	< 0.001
1–3 h	202 (39.8%)	79 (51.0%)		128 (27.7%)	92 (42.4%)	
> 3 h	43 (8.5%)	16 (10.3%)		14 (3.0%)	22 (10.1%)	
Nutrition (average (CI)^††^)						
Protein (%E)	14.04 (13.70–14.39)	13.92 (13.46–14.38)	0.573	13.96 (13.57–14.34)	13.97 (13.54–14.39)	0.967
Fat (%E)	27.56 (26.77–28.35)	27.87 (26.83–28.92)	0.537	28.62 (27.75–29.49)	29.13 (28.17–30.10)	0.267
Carbohydrate (%E)	56.82 (55.83–57.80)	56.64 (55.33–57.94)	0.774	56.02 (54.95–57.09)	55.53 (54.34–56.71)	0.389
Saturated fatty acids (%E)	9.00 (8.67–9.33)	9.30 (8.86–9.74)	0.148	9.35 (8.97–9.72)	9.56 (9.14–9.97)	0.293
n-3 fatty acids (%E)	1.04 (0.99–1.08)	1.01 (0.95–1.06)	0.265	1.08 (1.03–1.12)	1.10 (1.05–1.15)	0.363
n-6 fatty acids (%E)	4.86 (4.70–5.02)	4.81 (4.60–5.02)	0.590	5.10 (4.92–5.28)	5.14 (4.94–5.35)	0.635
Sodium (mg/1000 kcal)	2230.79 (2156.53–2305.05)	2218.11 (2119.69–2316.53)	0.789	2343.66 (2257.26–2430.05)	2357.79 (2261.95–2453.62)	0.759
Potassium (mg/1000 kcal)	1210.70 (1169.07–1252.33)	1180.08 (1124.92–1235.25)	0.249	1224.07 (1179.51–1268.63)	1213.28 (1163.86–1262.71)	0.649
Calcium (mg/1000 kcal)	353.47 (336.41–370.52)	356.25 (333.65–378.86)	0.798	358.14 (341.52–374.77)	360.83 (342.39–379.27)	0.761
Iron (mg/1000 kcal)	3.62 (3.51–3.73)	3.56 (3.41–3.70)	0.378	3.73 (3.61–3.86)	3.75 (3.61–3.89)	0.805
Vitamin D (μg/1000 kcal)	5.11 (4.69–5.54)	5.02 (4.46–5.58)	0.728	5.02 (4.55–5.50)	5.56 (5.03–6.09)	0.034
Vitamin K (μg/1000 kcal)	117.76 (109.90–125.62)	106.65 (96.23–117.06)	0.027	110.77 (101.51–120.02)	113.92 (103.66–124.18)	0.522
Vitamin B-1 (mg/1000 kcal)	0.41 (0.40–0.42)	0.40 (0.39–0.42)	0.621	0.40 (0.39–0.42)	0.40 (0.39–0.42)	0.757
Vitamin B-2 (mg/1000 kcal)	0.72 (0.69–0.74)	0.71 (0.68–0.75)	0.774	0.72 (0.69–0.75)	0.72 (0.69–0.75)	0.742
Folic acid (μg/1000 kcal)	148.54 (141.84–155.25)	143.24 (134.35–152.13)	0.215	152.77 (145.31–160.23)	149.59 (141.31–157.86)	0.422
Vitamin C (mg/1000 kcal)	52.52 (49.18–55.86)	51.49 (47.06–55.91)	0.628	55.60 (51.84–59.36)	55.13 (50.95–59.30)	0.813
Cholesterol (mg/1000 kcal)	164.64 (156.55–172.72)	168.48 (157.77–179.20)	0.456	174.07 (164.03–184.11)	178.87 (167.74–190.00)	0.369
Total dietary fiber (g/1000 kcal)	5.60 (5.40–5.79)	5.48 (5.22–5.74)	0.336	5.67 (5.44–5.89)	5.66 (5.41–5.91)	0.976

^†^*χ*^2^ tests for categorical variables and analysis of covariances (ANCOVA) for continuous variables, adjusting for age, BMI, and physical activity

^††^Adjusted mean and standard errors calculated by ANCOVA

Boys who used PC had smaller intake of potassium (*p* = 0.056), iron (*p*2 = 0.060), vitamin K (*p* = 0.029), and folic acid (*p* = 0.047). There was no difference in nutrient intake between girls who used PC and girls who did not use PC.

Boys who used MP consumed less vitamin K (*p* = 0.027). Girls who used MP were more likely to consume vitamin D (*p* = 0.034).

### Relationship between screen time and nutrient intake

The relationship between screen time and nutrient intake are shown in Tables 5, 6 and 7. The following nutrient intake remained correlated or tended to correlate with screen time.

**Table 5:** The relationship between television viewing and nutrient intake

	Boys (*N* = 664)			Girls (*N* = 682)		
	*β*	Lower CI to upper CI	*p* ^†^	*β*	Lower CI to upper CI	*p* ^†^
Protein (%E)	− 0.109	(− 0.184 to − 0.033)	0.005	− 0.085	(− 0.159 to − 0.009)	0.027
Fat (%E)	− 0.019	(− 0.096 to 0.058)	0.632	0.030	(− 0.045 to 0.107)	0.430
Carbohydrate (%E)	0.054	(− 0.024 to 0.131)	0.172	0.010	(− 0.066 to 0.085)	0.803
Saturated fatty acids (%E)	0.001	(− 0.076 to 0.077)	0.989	− 0.027	(− 0.102 to 0.048)	0.485
n-3 fatty acids (%E)	− 0.048	(− 0.124 to 0.029)	0.221	0.038	(− 0.038 to 0.113)	0.327
n-6 fatty acids (%E)	− 0.038	(− 0.110 to 0.034)	0.299	0.079	(0.004 to .154)	0.040
Sodium (mg/1000 kcal)	− 0.046	(− 0.110 to 0.023)	0.200	− 0.065	(− 0.126 to 0.005)	0.072
Potassium (mg/1000 kcal)	− 0.076	(− 0.158 to 0.000)	0.050	− 0.056	(− 0.129 to 0.018)	0.142
Calcium (mg/1000 kcal)	− 0.109	(− 0.189 to − 0.033)	0.005	− 0.175	(− 0.246 to − 0.098)	< 0.001
Iron (mg/1000 kcal)	− 0.084	(− 0.159 to − 0.009)	0.029	− 0.038	(− 0.112 to 0.037)	0.328
Vitamin D (μg/1000 kcal)	− 0.039	(− 0.111 to 0.035)	0.312	− 0.064	(− 0.139 to 0.011)	0.096
Vitamin K (μg/1000 kcal)	− 0.098	(− 0.170 to − 0.022)	0.011	− 0.028	(− 0.102 to 0.047)	0.474
Vitamin B-1 (mg/1000 kcal)	− 0.061	(− 0.142 to 0.015)	0.112	− 0.040	(− 0.113 to 0.034)	0.288
Vitamin B-2 (mg/1000 kcal)	− 0.076	(− 0.154 to − 0.001)	0.046	− 0.079	(− 0.152 to − 0.006)	0.035
Folic acid (μg/1000 kcal)	− 0.068	(− 0.149 to 0.009)	0.081	− 0.010	(− 0.084 to 0.064)	0.794
Vitamin C (mg/1000 kcal)	− 0.016	(− 0.097 to 0.064)	0.683	0.038	(− 0.036 to 0.112)	0.313
Cholesterol (mg/1000 kcal)	− 0.060	(− 0.138 to 0.016)	0.122	0.012	(− 0.062 to 0.085)	0.758
Total dietary fiber (g/1000 kcal)	− 0.074	(− 0.149 to 0.000)	0.051	− 0.025	(− 0.097 to 0.049)	0.514

^†^Multivariate linear regression analyses, adjusting for age, BMI, and physical activity

**Table 6:** The relationship between personal computer using and nutrient intake

	Boys (*N* = 664)			Girls (*N* = 680)		
	*β*	Lower CI to upper CI	*p* ^†^	*β*	Lower CI to upper CI	*p* ^†^
Protein (%E)	− 0.051	(− 0.131 to 0.029)	0.208	− 0.014	(− 0.095 to 0.066)	0.725
Fat (%E)	− 0.013	(− 0.093 to 0.067)	0.749	0.037	(− 0.044 to 0.119)	0.367
Carbohydrate (%E)	0.028	(− 0.053 to 0.109)	0.495	− 0.026	(− 0.108 to 0.055)	0.523
Saturated fatty acids (%E)	0.001	(− 0.079 to 0.080)	0.984	0.052	(− 0.029 to 0.133)	0.206
n-3 fatty acids (%E)	− 0.050	(− 0.130 to 0.029)	0.216	0.023	(− 0.058 to 0.105)	0.571
n-6 fatty acids (%E)	− 0.044	(− 0.119 to 0.030)	0.243	− 0.016	(− 0.097 to 0.065)	0.692
Sodium (mg/1000 kcal)	− 0.051	(− 0.118 to 0.021)	0.172	− 0.001	(− 0.071 to 0.070)	0.989
Potassium (mg/1000 kcal)	− 0.076	(− 0.162 to 0.003)	0.059	− 0.025	(− 0.104 to 0.055)	0.538
Calcium (mg/1000 kcal)	− 0.037	(− 0.120 to 0.043)	0.357	0.009	(− 0.072 to 0.089)	0.836
Iron (mg/1000 kcal)	− 0.075	(− 0.154 to 0.003)	0.061	− 0.015	(− 0.096 to 0.066)	0.714
Vitamin D (μg/1000 kcal)	0.028	(− 0.049 to 0.104)	0.486	0.045	(− 0.035 to 0.126)	0.270
Vitamin K (μg/1000 kcal)	− 0.085	(− 0.162 to − 0.007)	0.033	− 0.009	(− 0.089 to 0.072)	0.834
Vitamin B-1 (mg/1000 kcal)	− 0.036	(− 0.120 to 0.045)	0.370	− 0.047	(− 0.126 to 0.032)	0.240
Vitamin B-2 (mg/1000 kcal)	− 0.041	(− 0.122 to 0.038)	0.299	− 0.004	(− 0.083 to 0.075)	0.918
Folic acid (μg/1000 kcal)	− 0.078	(− 0.164 to 0.001)	0.053	− 0.026	(− 0.107 to 0.054)	0.515
Vitamin C (mg/1000 kcal)	− 0.044	(− 0.131 to 0.037)	0.272	− 0.019	(− 0.099 to 0.060)	0.633
Cholesterol (mg/1000 kcal)	0.010	(− 0.070 to 0.091)	0.805	0.034	(− 0.045 to 0.113)	0.399
Total dietary fiber (g/1000 kcal)	− 0.052	(− 0.132 to 0.026)	0.186	− 0.004	(− 0.083 to 0.074)	0.916

^†^Multivariate linear regression analyses, adjusting for age, BMI, and physical activity

**Table 7:** The relationship between mobile phone using and nutrient intake

	Boys (*N* = 663)			Girls (*N* = 679)		
	*β*	Lower CI to upper CI	*p* ^†^	*β*	Lower CI to upper CI	*p* ^†^
Protein (%E)	− 0.023	(− 0.102 to 0.057)	0.575	0.001	(− 0.081 to 0.084)	0.976
Fat (%E)	0.027	(− 0.052 to 0.108)	0.496	0.048	(− 0.035 to 0.133)	0.254
Carbohydrate (%E)	− 0.014	(− 0.094 to 0.067)	0.738	− 0.037	(− 0.121 to 0.046)	0.378
Saturated fatty acids (%E)	0.060	(− 0.019 to 0.139)	0.138	0.046	(− 0.037 to 0.129)	0.277
n-3 fatty acids (%E)	− 0.043	(− 0.123 to 0.036)	0.284	0.039	(− 0.044 to 0.122)	0.355
n-6 fatty acids (%E)	− 0.019	(− 0.093 to 0.056)	0.626	0.021	(− 0.061 to 0.105)	0.610
Sodium (mg/1000 kcal)	− 0.012	(− 0.081 to 0.058)	0.751	0.012	(− 0.061 to 0.083)	0.767
Potassium (mg/1000 kcal)	− 0.047	(− 0.132 to 0.033)	0.242	− 0.020	(− 0.101 to 0.062)	0.636
Calcium (mg/1000 kcal)	0.009	(− 0.072 to 0.091)	0.823	0.013	(− 0.069 to 0.096)	0.754
Iron (mg/1000 kcal)	− 0.036	(− 0.115 to 0.043)	0.370	0.009	(− 0.074 to 0.091)	0.832
Vitamin D (μg/1000 kcal)	− 0.013	(− 0.089 to 0.063)	0.736	0.088	(0.006 to 0.171)	0.036
Vitamin K (μg/1000 kcal)	− 0.089	(− 0.166 to − 0.011)	0.026	0.026	(− 0.056 to 0.107)	0.536
Vitamin B-1 (mg/1000 kcal)	− 0.020	(− 0.103 to 0.062)	0.624	− 0.014	(− 0.095 to 0.067)	0.737
Vitamin B-2 (mg/1000 kcal)	− 0.011	(− 0.091 to 0.068)	0.781	− 0.015	(− 0.095 to 0.066)	0.722
Folic acid (μg/1000 kcal)	− 0.050	(− 0.135 to 0.030)	0.215	− 0.035	(− 0.117 to 0.047)	0.398
Vitamin C (mg/1000 kcal)	− 0.019	(− 0.103 to 0.064)	0.643	− 0.012	(− 0.093 to 0.070)	0.779
Cholesterol (mg/1000 kcal)	0.031	(− 0.049 to 0.111)	0.443	0.037	(− 0.043 to 0.119)	0.360
Total dietary fiber (g/1000 kcal)	− 0.040	(− 0.119 to 0.038)	0.314	− 0.002	(− 0.083 to 0.078)	0.954

^†^Multivariate linear regression analyses, adjusting for age, BMI, and physical activity

In boys, longer TV viewing times correlated with a lower intake of protein (*β* = − 0.109, *p* = 0.005), potassium (*β* = − 0.076, *p* = 0.050), calcium (*β* = − 0.109, *p* = 0.005), iron (*β* = − 0.084, *p* = 0.029), vitamin K (*β* = − 0.098, *p* = 0.011), vitamin B-2 (*β* = − 0.076, *p* = 0.046), and total dietary fiber (*β* = − 0.074, *p* = 0.051). In girls, longer TV viewing times correlated with a lower intake of protein (*β* = − 0.085, *p* = 0.027), sodium (*β* = − 0.065, *p* = 0.072), calcium (*β* = − 0.175, *p* < 0.001), vitamin D (*β* = − 0.064, *p* = 0.096), and vitamin B-2 (*β* = − 0.079, *p* = 0.035). Longer TV viewing times correlated with a higher intake of n-6 fatty acids (*β* = 0.079, *p* = 0.040).

PC use was related or tended to be related to the lower intake of potassium (*β* = − 0.076, *p* = 0.059), iron (*β* = − 0.075, *p* = 0.061), vitamin K (*β* = − 0.085, *p* = 0.033), and folic acid (*β* = − 0.078, *p* = 0.053) in boys, but not in girls.

A relationship was observed between MP use and a lower intake of vitamin K (*β* = − 0.089, *p* = 0.026) in boys, and MP use and a higher intake of vitamin D (*β* = 0.088, *p* = 0.036) in girls.

## Discussion

While many studies have investigated relationships among sedentary behaviors and diet, few have examined those among sedentary behaviors and nutrient intake in children and adolescents. Each of these health behaviors is an independent risk factor for chronic diseases, and thus, studies are required in order to establish whether these behaviors need to be targeted independently or jointly. Since technology-based sedentary behaviors may change quickly, relationships with new types of sedentary behaviors need to be examined. Therefore, the purpose of the present study was to elucidate the relationship between screen time and nutrient intake in children and adolescents. The results obtained showed a relationship between longer TV viewing times and less protein, minerals, vitamins, and total dietary fiber intake. Relationship between PC use in boys and less minerals and vitamins was also shown. On the contrary, PC use in girls and MP use was not strongly implicated in these nutrient intakes. Our results suggest that some types of screen time and nutrient intake do not occur in isolation.

A previous study that analyzed the combined data of boys and girls noted a negative relationship between TV viewing and protein intake [18], whereas this relationship was not detected in three other studies [16, 17]. In the present study, we found negative relationships in boys and girls. TV viewing was negatively associated with calcium intake in three out of five previous studies [15, 17, 18]. We found that TV viewing negatively correlated with calcium intake in boys and girls. Although a previous study reported no correlation between TV viewing and iron intake [19], we found this relationship in boys. Previous studies reported a negative relationship between TV viewing and total dietary fiber [15, 17, 19–21]. This finding is consistent with the present results obtained from boys [22–24]. In addition to these results, negative correlations were observed between TV viewing and potassium, vitamin K, and vitamin B-2 in boys, and sodium, vitamin D, and vitamin B-2 in girls. Positive correlation was also found between TV viewing and n-3 fatty acids in girls.

A meta-analysis revealed that the relationship between weight status and screen time in young individuals was weak or inconsistent [25]. Weak or inconsistent relationship between screen time and weight status may be attributed to the moderating effects of nutrient intake. In other words, weight status may be related to nutritional consumption from screen time, resulting in inconsistencies in findings when investigating screen time and weight status only. A high frequency of the consumption of food in front of the TV has the potential to have an impact on the energy balance and, ultimately, body weight [15]. Sugar-sweetened beverages have been implicated in weight gain [26] and may be one of the mechanisms linking screen time with obesity.

Several mechanisms have been proposed to explain the relationship between screen behavior and dietary intake. One possible explanation is that children and adolescents with more screen time may be less physically active. This reduced PA may lead to a less healthy nutrient intake. However, we found a relationship between TV viewing and nutrient intake even after adjustments for PA. Another explanation is that screen users are exposed to numerous advertisements that may influence the type of food consumed [27]. A previous study on advertising and programming content reported the presence of frequent advertisements for “fast food, sugar-sweetened beverages, and other nutrient-poor calorically dense snacks along with a lack of content for low-calorie, nutrient-dense foods such as fruit and vegetables” [28]. Observed negative correlation between TV viewing and sodium intake may be due to more intake of such nutrient-poor foods instead of staple foods, main dishes, or side dishes which contain high levels of sodium in Japan. Furthermore, it has been hypothesized that individuals eat more while watching TV because they are distracted [29]. “Distracted eating” is “the dominance of the automatic system (over the reflective one) when making decisions pertaining to eating during TV viewing; that is, people are more likely to make emotional and uncontrolled decisions about eating and are less exposed to cues that would limit food intake when distracted by screen time” [29]. Experimental studies [30, 31] have shown that watching TV while eating may cause a distraction. Future studies are needed in order to elucidate the underlying mechanisms.

Due to the recent decrease in TV screen time and increase in PC and MP screen times among young individuals, the relationship between PC and MP use and nutrient intake differed from that between TV viewing and nutrition intake after adjustments for age, BMI, and PA. In girls, a positive correlation was observed between MP use and vitamin D intake, unexpectedly. MP use may have some good influence on nutrient intake in girls. However, we found such correlation only in vitamin D. We need more detailed analysis to unravel this finding. The relationship between PC use in girls and MP use and nutrient intake (Tables 2, 3, and 4) may be attributed to PA. PC use in girls and MP use may not have a similar effect as TV viewing.

From the perspective of public health, our results demonstrate that children and adolescents need to reduce the amount of time they spend watching TV as well as the frequency of eating in front of the TV screen in order to reduce health risks. Besides, girls need to be careful not to increase the amount of sodium they take. Boys need to reduce the time spent for using PC.

The strength of the present study is that we conducted a complete survey on children aged between 6 and 15 years old in one town. We also collected questionnaires at a high response rate. These strengths allowed us to examine the relationship between screen time and nutrient intake, excluding selection biases, especially non-respondent bias or volunteer bias, as much as possible.

The limitations of the present study were as follows. This study relied on self-reported screen times, which are only proxies of sedentary times and are subject to recall bias, even though self-report methods of quantifying screen time have been shown to have acceptable reliability and validity in children [32]. Furthermore, despite the use of a validated and detailed one-month food record, we cannot exclude possible measurement errors in nutrition. Therefore, adjustments for energy intake are suggested in order to reduce bias when examining the relationship between individuals’ dietary intakes and other health-related outcomes. We adhered to this in the present study. Moreover, the BDHQ 10y and 15y do not include the calculation for supplements, which could influence calculation results of nutrient values. Another limitation is that the study design was cross-sectional in nature; therefore, the causality and directionality of relationships cannot be assessed. Future studies need to be longitudinal in design. In addition, we did not consider the similarities between participants from the same family.

## Conclusions

The present results revealed that children and adolescents with longer TV viewing times have less protein, minerals, vitamins, and total dietary fiber. It was also revealed that boys with PC use have less minerals and vitamins. These results support the need to design intervention programs that focus on decreasing TV viewing times in both sexes and PC use in boys while encouraging adherence to dietary guidelines among children and adolescents. This is crucial because lifestyle habits are formed at an early age and are likely to track into adulthood.

## Abbreviations

BDHQ: Brief self-administered dietary history questionnaire
BMI: Body mass index
CI: Confidence interval
MP: Mobile phone
PA: Physical activity
PC: Personal computer
SD: Standard deviation
SPSS: Statistical Package for Social Science
TV: Television
